# Structure and sequence evolution in the pennycress (*Thlaspi arvense*) pangenome

**DOI:** 10.1111/nph.71111

**Published:** 2026-04-02

**Authors:** Kevin A. Bird, Joanna L. Rifkin, Chloee M. McLaughlin, Avril M. Harder, Pawan Basnet, Ella Katz, Tomáš Brůna, Kerrie Barry, LoriBeth Boston, Christopher Daum, Jie Guo, Anna Lipzen, Christopher Plott, Jerry W. Jenkins, Rachel Walstead, Shanmugam Rajasekar, Jayson Talag, Katherine Frels, Kathleen Greenham, Shelby Ellison, Jane Grimwood, Jeremy Schmutz, Patrick P. Edger, J. Chris Pires, John T. Lovell, Daniel J. Kliebenstein

**Affiliations:** ^1^ Department of Trait Diversity and Function Royal Botanic Gardens, Kew Richmond, Surrey TW9 3AE UK; ^2^ Department of Plant Sciences University of California at Davis Davis CA 95616 USA; ^3^ HudsonAlpha Institute for Biotechnology Huntsville AL 35806 USA; ^4^ Department of Plant and Agroecosystem Sciences University of Wisconsin, Madison Madison WI 53706 USA; ^5^ US Department of Energy Joint Genome Institute Lawrence Berkeley National Laboratory Berkeley CA 94720 USA; ^6^ Arizona Genomics Institute University of Arizona Tucson AZ 85721 USA; ^7^ Department of Agronomy and Horticulture University of Nebraska Lincoln NE 68583 USA; ^8^ Department of Plant and Microbial Biology University of Minnesota St. Paul MN 55108 USA; ^9^ Department of Horticulture MI State University East Lansing MI 48824 USA; ^10^ Department of Soil and Crop Sciences CO State University Fort Collins CO 80523 USA

**Keywords:** centromere, Pangenome, resistance genes, structural variation, *Thlaspi arvense*

## Abstract

- Eukaryotic genomes harbor many forms of variation, including nucleotide diversity and structural polymorphisms, which experience natural selection and contribute to genome evolution and biodiversity. Harnessing this variation for agriculture hinges on our ability to detect, quantify, catalog, and deploy genetic diversity.
- Here, we explore seven complete genomes of the emerging biofuel crop pennycress (*Thlaspi arvense*) drawn from across the species' current genetic diversity to catalog variation in genome structure and content.
- Across this new pangenome resource, we find contrasting evolutionary modes in different genomic zones. Gene‐poor, repeat‐rich pericentromeric regions experience frequent rearrangements, including repeated centromere repositioning. By contrast, conserved gene‐dense chromosome arms maintain large‐scale synteny across accessions even in fast‐evolving NOD‐like receptor immune genes, where microsynteny breaks down across species, but gene cluster positioning macrosynteny is maintained.
- Our findings highlight that multiple elements of the genome experience dynamic evolution that conserves functional content on the chromosome scale but allows repositioning and presence–absence variation on a local scale. This diversity is invisible to classical reference‐based strategies and highlights the strength and utility of pangenomic resources. These results provide a valuable case study of rapid genomic structural evolution within a species and powerful resources for crop development in an emerging biofuel crop.

**Table:** 

	Contents	
	Summary	2723
I.	Introduction	2724
II.	Materials and Methods	2725
III.	Results	2729
IV.	Discussion	2735
V.	Conclusions	2737
	Acknowledgements	2737
	References	2738

## I. Introduction

Genetic diversity in crops allows for resilience and sustainability in agricultural systems. In established crops, DNA sequence variation that underlies quantitative genetic variation is leveraged to develop trait combinations and cultivars that respond to changing pests, pathogens, and climates, and to minimize the environmental impact of agriculture. There has also been considerable effort to develop new crops from wild species that already harbor stress tolerance and novel end‐use trait variation (e.g. seed oil chemistry). However, such *de novo* domestication is typically constrained by limited genetic variation within traits needed for efficient growth and harvesting in agricultural systems (e.g. loss of shattering and low seed dormancy). These constraints can be circumvented by genome editing or mutant screens (Lemmon *et al*., 2018; Zsögön *et al*., 2018; Gasparini *et al*., 2021; Gautam *et al*., 2026), but these approaches require species amenable to transformation with genomes that are tractable for investigations of genetic variation underlying the traits of interest (e.g. loss‐of‐function, gain‐of‐function, and dosage change). These requirements become more onerous with greater evolutionary distance to model systems (Roeder *et al*., 2025). Thus, improvement of domesticated species, and successful development of new crop species, hinge on our ability to detect, quantify, catalog, and deploy genetic diversity.

Genome sequences and associated resources allow for surveys of genetic diversity, identification of valuable gene pools, and discovery of causal loci that underlie traits of interest in crops and crop wild relatives, with the ultimate goal of transferring genetic variation among gene pools to develop new elite cultivars. However, the success of these transfers depends on the genomic context of sequence variation that underlies traits of interest. In an ideal scenario, traits are unlinked and in high‐recombination regions, allowing desirable alleles to be stacked without interference from deleterious variants. However, gene density and recombination rate vary predictably in most plant genomes, sorting most sequence into two ‘compartments’: ‘pericentromeres’ are low recombination highly repetitive regions adjacent to centromeres, while ‘chromosome arms’ typically harbor most of the genes and recombination events. Such covariance between chromosome architecture and recombination landscape impacts the evolutionary dynamics of genetic diversity, as well as its accessibility to selection agents. For example, pericentromeric sequences often have weaker signatures of purifying selection than chromosome arms, likely due to lower recombination rates and increased effects of linkage drag (Chen *et al*., 2022). Such relaxed selection pressures mean that pericentromeres often harbor greater sequence and structural diversity than subtelomeric chromosome arms (Kawabe *et al*. 2004; Hall *et al*., 2006; Flowers *et al*., 2012), sometimes to such an extent that they are the proximate cause of reproductive barriers between species (Ma & Zhu, 2025). Despite millennia of selection and intercrossing, most domesticated species exhibit variable degrees of reproductive isolation, which can constrain efforts to introgress traits between highly diverged gene pools (e.g. sorghum and rice). Therefore, it is likely that contemporary efforts to domesticate plant species and develop new crops will confront challenges related to reproductive isolation, possibly due to structural evolution among pericentromeres.

Pennycress (*Thlaspi arvense* L.; 2*n* = 2*x* = 14), an annual weedy species in the *Brassicaceae* family native to Eurasia, offers an excellent system to explore functional and evolutionary consequences of pangenomic variation during *de novo* domestication. Several attractive agronomic and genomic attributes have made pennycress a target for domestication and improvement for use as a winter cover crop and oil feedstock (Boateng *et al*., 2010; Moser, 2012; Fan *et al*., 2013). Agronomically, the winter hardiness and short life cycle of pennycress make it well‐suited to double‐crop systems that can increase productivity and foster sustainable agriculture by repairing soil health, reducing erosion, providing pollinator food sources, and improving crop yields (Phippen & Phippen, 2012; Del Gatto *et al*., 2015; Johnson *et al*., 2015; Weyers *et al*., 2019, 2021; Basnet & Ellison, 2024). Pennycress has a medium‐sized diploid genome (*c*. 526 Mb based on flow cytometry; Lysak *et al*., 2008) and a predominantly one‐to‐one gene ratio with the model plant *Arabidopsis thaliana* (Arabidopsis), both of which aid its domestication and improvement by accelerating identification of candidate orthologs with functional effects on key traits (Chopra *et al*., 2018, 2020; Roeder *et al*., 2025). Both classical breeding and targeted gene editing efforts are further bolstered by the predominantly self‐fertilizing habit of pennycress and its amenability to floral dip transformation (Mulligan & Kevan, 1973; McGinn *et al*., 2019). Despite these advantages enabling *de novo* domestication, pennycress's limited genetic diversity and susceptibility to biotic pests and pathogens, such as soybean cyst nematode, aphids, black rot, black spot disease, and clubroot disease are persistent barriers to widespread adoption (Basnet & Ellison, 2024).

Research and development of pennycress has greatly benefited from the recently improved reference genome (Dorn *et al*., 2015; García Navarrete *et al*., 2022; Nunn *et al*., 2022), which has supported short‐read resequencing of pennycress diversity panels to characterize genetic diversity in the species across its range. Populations from Western Europe and North America have low levels of genetic distance, as expected given that pennycress is believed to have been introduced to North America by European immigration within the last 250 yr (Frels *et al*., 2019; Nunn *et al*., 2022). In its native range, pennycress exhibits minimal population structure between Western European and East Asian populations. However, multiple studies have identified populations in Armenia as genetically distinct from all other pennycress populations, showing high levels of genetic distance and unidirectional gene flow out of but not into Armenia (Frels *et al*., 2019; Nunn *et al*., 2022; Contreras‐Garrido *et al*., 2024; Wu *et al*., 2025). The single reference genome also enabled transposable element (TE) surveys, creating a list of transposon insertion polymorphisms that may be harnessable by scientists and breeders to accelerate crop improvement (Nunn *et al*., 2022; Contreras‐Garrido *et al*., 2024). While existing resources have identified candidate resistance loci through genome‐wide association studies (Galanti *et al*., 2024), characterizing resistant accessions and incorporating them into breeding programs requires a deeper understanding of genetic diversity in the species.

Improvements in sequencing technology have reduced the cost and effort needed to construct reference‐quality genomes, particularly in plants, which have historically been difficult to sequence because of large genomes, whole‐genome duplications, extensive repeats, and chromosomal rearrangements. Thanks to these improvements, collections of diverse reference genomes for a single species, known as pangenomes, are now available more broadly across the tree of life. Pangenome datasets enable better surveys of genetic variation, revealing previously hidden gene presence–absence variation (PAV) and structural variation shaping adaptation and gene flow across populations (Bayer *et al*., 2020; Katz *et al*., 2021; Zhang *et al*., 2021, Zhou *et al*., 2022; Schreiber *et al*., 2024). These pangenomic catalogs of sequence variation improve our ability to identify the genetic bases of traits and apply molecular breeding approaches like *de novo* domestication (Gasparini *et al*., 2021; Zhou *et al*., 2022; Jin *et al*., 2023; Liu *et al*., 2024). This is particularly the case for complex loci, such as the NOD‐Like Receptor (NLR) pathogen resistance genes, which often vary dramatically within species because of rapid duplication and loss (Teasdale *et al*., 2025).

To begin developing pangenomic resources for pennycress, we present seven complete, chromosome‐scale genomes sampled across the genetic diversity of pennycress. Our pangenomic analyses found strong genomic compartmentalization in pennycress, in which genome architecture defines distinct evolutionary regimes that differ in their prevalence of structural variation. We summarize this pattern as a ‘two‐speed’ genome organization for structural evolution: gene‐rich chromosome arms exhibited high levels of synteny and low levels of presence/absence variation among accessions, whereas gene‐poor highly repetitive peri‐ and centromeric regions contained extensive genomic rearrangement, especially between Armenian and non‐Armenian accessions. These genomic rearrangements included centromeric movement within the species, and population genomic analyses suggested these rearrangements may partially limit gene flow among populations. Finally, we facilitated potential efforts toward disease‐resistance breeding by developing a catalog of high‐confidence NLR genes and identified a subset of NLR loci as highly variable. Comparative genomic analyses revealed that NLR gene clusters tend to be positionally conserved across significant evolutionary distances between species, despite high diversity and gene turnover such that particular NLR genes are unlikely to be orthologous between species.

## II. Materials and Methods

### 1. Plant material, DNA, and RNA extraction

High molecular weight DNA was extracted from leaf material of seven genotypes of *Thlaspi arvense* L. (2*n* = 2*x* = 14) using the protocol of Doyle & Doyle (1987) with minor modifications. Flash‐frozen biomass was ground to a fine powder in a frozen mortar with liquid nitrogen followed by very gentle extraction in 2% CTAB buffer (that included proteinase K, PVP‐40 and beta‐mercaptoethanol) for 30 min to 1 h at 50°C. After centrifugation, the supernatant was transferred to a new tube, treated with 200 μl 50 mM PSMF for 10 min at room temperature, and then gently extracted twice with 24 : 1 Chloroform : Isoamyl alcohol. The upper phase was transferred to a new tube and 1/10^th^ volume 3 M Sodium acetate was added, gently mixed, and DNA precipitated with iso‐propanol. DNA precipitate was collected by centrifugation, washed with 70% ethanol, air dried for 5–10 min and dissolved thoroughly in elution buffer at room temperature followed by RNAse treatment. DNA purity was measured with Nanodrop, DNA concentration measured with Qubit HS kit (Invitrogen), and DNA size was validated by Femto Pulse System (Agilent).

### 2. DNA sequencing and genome assembly

We employed a whole‐genome shotgun sequencing strategy for the seven *T. arvense* genomes. Sequencing libraries were constructed for all accessions for PacBio HiFi sequencing using circular consensus sequencing (CCS) mode. We used a Diagenode Megaruptor 3 for DNA shearing, prepared libraries using SMRTbell Template Prep Kit 2.0 kits, and sized our libraries using a SAGE ELF instrument with a 1–18‐kb cassette. Illumina polishing libraries were prepared for all accessions using Illumina TruSeq PCRfree library prep kits (catalog no.: 20015962). In addition, Omni‐C libraries were prepared for extra scaffolding using Dovetail Omni‐C kits (catalog no.: 21005) for MN106 and Ames 32 873.

We sequenced our PacBio libraries on the SEQUEL II platform using one to three single molecule, real‐time (SMRT) cells with V2 chemistry, and our Illumina libraries on the NovoSeq 6000 platform. All sequencing was performed at the HudsonAlpha Institute in Huntsville, AL, USA. All accessions were sequenced with PacBio coverage depths between 70.23 and 88.35× and read lengths averaging between 15 194 and 18 898 bp (Supporting Information Dataset S1). For polishing, all accessions were sequenced with Illumina (2 × 150, 400‐bp insert) coverage depths between 49.1× and 54.7×. We also sequenced 2 × 150‐bp Omni‐C libraries for MN106 and Ames 32 873.

Genomes were assembled using HiFiAsm v.0.16.1‐r375 (Cheng *et al*., 2021, 2022) with Hi‐C integration (‐h1 and ‐h2) used for MN106 and Ames 32 873. For MN106 and Ames 32 873, contigs were aligned and oriented using Hi‐C reads with the JUICER pipeline. For all other genomes, contig positions were finalized using a total of 11 295 unique, nonrepetitive, nonoverlapping 1‐KB syntenic markers from the v.3.0 MN106 genome aligned to the RACON‐polished assembly (Vaser *et al*., 2017) using BLAT (Kent, 2002). The initial assemblies consisted of between 11 and 23 contigs, which were combined using between five and eight joins into seven scaffolds. Every genome contained at least one single‐contig chromosome, and no chromosome consisted of more than six contigs. No contig breaks were necessary for any of the genomes assemblies. In a final round of polishing, Illumina sequence was used to correct homozygous single‐nucleotide polymorphisms (SNPs) and indels with a pipeline composed of bwa mem (v.2.2.1; Li & Durbin, 2009) and GATK's UnifiedGenotyper tool (v.3.7; McKenna *et al*., 2010). Fewer than 21 SNPs and 600 indel errors were corrected for all genomes except Tibet 33, in which over 1200 SNPs and over 4300 indels were corrected.

We assessed the completeness of the euchromatic portion of the genomes by aligning *T. arvense* V1 annotated genes to each assembly with BLAT, retaining the longest alternative splicing variant and excluding alignments with < 90% identity or < 85% coverage. Completeness estimated this way was 97.86 for the highly divergent Ames 32 879 genome but between 99.04 and 99.33 for all other accessions.

### 3. RNA sequencing and genome annotation

For all seven accessions, sequencing libraries were prepared for PacBio Iso‐Seq RNA sequencing (Dataset S2). We synthesized full‐length cDNA with NEBNext Single Cell/Low Input cDNA Synthesis & Amplification Module kit (catalog no.: E6421) by amplifying first‐strand cDNA with NEBNext High‐Fidelity 2× PCR Master Mix (catalog no.: M0541) with barcoded cDNA PCR primers for 11–14 cycles. We then purified cDNA using 1× AMPure PB beads (catalog no.: 100–265‐900; PacBio) for nonsize selection or BluePippin (Sage Science, Beverly, MA, USA) for 2–10‐kb size selection. Sizes were pooled in equimolar ratios according to the PacBio Multiplexing Calculator worksheet (PacBio, Menlo Park, CA, USA). We next end‐repaired, A‐tailed, and ligated overhang nonbarcoded adaptors to our samples using SMRTbell Express 2.0 Kit (catalog no.: 100‐938‐900; PacBio). PacBio Sequencing primer was annealed to the SMRTbell template library. Sequencing polymerase was bound to samples using Sequel II Binding kit 2.0 (catalog no.: 101–789‐500; PacBio). Finally, we sequenced the libraries on a PacBio Revio sequencer. Sequencing used SMRT Link 10.2, sample‐dependent sequencing primer, 8M v.1 SMRT cells, and v.2.0 sequencing chemistry with 1 × 1800 sequencing movie run times (PacBio).

We also prepared Illumina sequencing libraries. Plate‐based sample prep was performed using Illumina TruSeq‐stranded mRNA HT sample prep kits (catalog no.: 20020595) on PerkinElmer Sciclone NGS robotic liquid handling system (Revvity, Waltham, MA, USA) according to Illumina's user guide (https://support.illumina.com/sequencing/sequencing_kits/truseq‐stranded‐mrna.html). We started with 1000 ng per sample and amplified for eight samples of PCR. Libraries were quantified with KAPA Biosystems next‐generation sequencing library qPCR kits (catalog no.: 07980140001; Roche) and run on a Roche LightCycler 480 real‐time PCR instrument (Roche Diagnostics, Indianapolis, IN, USA). Samples were sequenced on an Illumina NovaSeq sequencer using NovaSeq XP V1.5 reagent kits (Illumina, San Diego, CA, USA), S4 flowcell, following a 2 × 151 indexed run recipe.

We annotated each genome in two rounds.

#### Round 1

In the first round, we generated transcriptome assemblies for each genome using 2 × 150 bp Illumina RNA‐seq reads with PERTRAN (Lovell *et al*., 2018), which uses GSNAP (Wu & Nacu, 2010) for genome‐guided transcriptome assembly before validating, realigning, and correcting alignments and building a splice alignment graph. We then used a genome‐guided correction pipeline to collapse and correct PacBio Iso‐Seq CCSs to obtain putative full‐length transcripts. We aligned CCS reads to the genome with GMAP (Wu & Watanabe, 2005; Wu *et al*., 2016), corrected small indels in splice junctions, and clustered alignments with all introns ≥ 95% overlapping. The Program to Assemble Spliced Alignments (PASA) (Haas *et al*., 2003) was then applied to merge the Illumina‐based and PacBio‐based transcriptome assemblies.

We then created a *de novo* repeat library from repeats predicted by RepeatModeler2 (Flynn *et al*., 2020) from the *T. arvense* MN106 v.4.0 genome assembly, specifically for use in the gene annotation pipeline (repeats were also annotated independently on the finalized assemblies with panEDTA; to be described later). Repeats were restricted to those with significant protein‐coding domains as identified by InterProScan (Jones *et al*., 2014) with the Pfam (Mistry *et al*., 2021) and PANTHER (Mi *et al*., 2019) databases. This library was used to soft‐mask each genome with RepeatMasker (Smit *et al*., 2013–2015).

To identify putative gene loci, we integrated our transcript assembly alignments with EXONERATE (Slater & Birney, 2005) alignments of proteins from diverse plants (*Eutrema salsugineum*, *Capsella rubella*, *Arabidopsis thaliana*, *Rorippa islandica*, *Myagrum perfoliatum*, *Caulanthus amplexicaulis, Cleome violacea, Glycine max, Vitis vinifera, Liriodendron tulipifera, Brassica rapa*, *Malcolmia maritima*, *Cleomella arborea*, *Capparis spinosa, Schrenkiella parvula*, *Gossypium raimondii*, *Populus trichocarpa*, *Sorghum bicolor*, *Oryza sativa*, and *Beta vulgaris*) and the Swiss‐Prot release 2022_04 of eukaryote proteomes to the repeat‐soft‐masked genome. We allowed up to 2‐kb extension on both ends, except when that extended into another locus on the same strand. We used the homology‐based predictors FGENESH+ (Salamov & Solovyev, 2000) and FGENESH_EST (which uses expressed sequence tags rather than open reading frames to compute splice sites and introns), EXONERATE, PASA assembly open reading frames (ORFs; in‐house homology constrained ORF finder), and AUGUSTUS (Stanke *et al*., 2006) trained on the high‐confidence PASA assembly ORFs and with intron hints from short‐read alignments. We selected the best‐scored predictions from each locus using multiple positive factors, which included expressed sequence tags and protein support, and the negative factor of overlap with repeats. We improved these predictions by using PASA to add UTRs, correct splicing, and add alternative transcripts.

We then performed protein homology analysis, comparing the PASA‐improved gene models to the diverse proteomes above, resulting in a Cscore (protein BLASTP score ratio to the mutual best hit BLASTP score) and protein coverage (percentage of protein aligned to the best of homologs). Transcripts with Cscore and protein coverage ≥ 0.5 were retained, as were transcripts covered by ESTs. However, gene models whose CDS overlapped repeats by more than 20% were retained only with Cscore ≥ 0.9 and protein coverage ≥ 0.7. Retained gene models were subjected to Pfam analysis, and models without strong transcriptome and homology support with more than 30% overlap of Pfam TE domains were also removed.

#### Round 2

After first‐round genome annotation, each genome was hard‐masked with its own high‐confidence (complete, transcriptome‐ and homology‐supported) gene models. The hard‐masked genomes were then aligned using BLASTX and EXONERATE against high‐confidence peptides from the other six genomes to make EXONERATE gene predictions. The resulting gene models were scored with BLASTP using homology proteomes. If the new models either (1) exhibited higher homology support and were not contradicted by transcriptome evidence or (2) were situated at loci without first‐round gene models, they were used to replace the original models (Dataset S2). We manually removed any gene models that met any of the following criteria: was incomplete, had low homology support and lacked full transcriptome support, had short single exons (< 300 bp CDS) without protein domains or expression support, or was repetitive and lacked strong homology support.

### 4. Centromere, telomere, and transposable element identification

We identified centromeres using TRASH v.1.2 (Wlodzimierz *et al*., 2023a) with default settings. We also identified centromeric regions using CentIER (Xu *et al*., 2024) without including our gene annotations because of gff format incompatibilities. In general, overlap between the regions identified with CentIER and the satellites located by TRASH was incomplete (Fig. S1). Since TRASH had successfully predicted a sequence also validated by fluorescent *in‐situ* hybridization (FISH), we therefore focused on the TRASH results for downstream analyses. We compared sequence similarity of putative satellite sequences using ClustalW (Thompson *et al*., 1997).

With custom R scripts, we located the largest block of putative satellite repeats identified by TRASH that were within 100 kb of each other in each genome (regions are shown in Fig. S2). We then inspected the structures of these regions, buffered by 1 Mb in either direction, when aligned to themselves using alignments generated with the nucmer command in MUMMER and plotted in R (Marçais *et al*., 2018). For all chromosomes in all genomes with the exception of Chromosome 6 in MN106, the structure we observed was consistent with expectations (Fig. S3). However, for Chromosome 6 in MN106, the second largest contiguous block of satellite repeats showed a structure more consistent with a centromeric region.

We annotated telomeres by clustering of putative plant telomeric repeats. We also performed a comprehensive annotation of TEs by first annotating each genome's RNA and DNA elements independently and then re‐annotating them with a consolidated repeat library, using the panEDTA in EDTA2 v.2.2.1 (Ou *et al*., 2019, 2024).

### 5. Synteny analysis, structural variant identification, and pangenome graph construction

We used Orthofinder v.2 (Emms & Kelly, 2019) to compare our new MN106 gene models with the v2 gene models, downloaded from the NCBI (accession no.: GCA_911865555.2), to provide a translational resource between versions (Dataset S4).

We identified general patterns of synteny and gene conservation within our *T. arvense* accessions and in related species in the Brassicaceae using GENESPACE v.1.3.1 (Lovell *et al*., 2022). For GENESPACE analyses, we identified regions of gene synteny between all seven *T. arvense* accessions and the Phytozome (Goodstein *et al*., 2012) repository versions of *Arabidopsis thaliana* (v. TAIR10/TAIR11), *A. lyrata* (v.2.1), and *Brassica rapa* (v. ssp. trilocularis R500 v.2.1) genomes. Syntenic blocks from GENESPACE and phylogenetic hierarchical orthogroups from Orthofinder v.2.5.5 (Emms & Kelly, 2019) run within GENESPACE were used in downstream analyses.

To identify and classify structural variation, each genome was aligned to the MN106 reference using minimap2 (v.2.26; Li, 2021) with asm5 preset options. The resulting alignments were filtered in R (v.4.4.1; R Core Team, 2024) to retain segments ≥ 1 kb in length with ≥ 80% sequence identity and MAPQ ≥ 2, and SyRI (v.1.6.3; Goel *et al*., 2019) was used to classify these alignments as syntenic regions or structural rearrangements. We constructed chromosome‐level pangenome graphs with Minigraph‐Cactus (v.2.9.0; Hickey *et al*., 2024) using default settings and MN106 as the primary reference. In the last step of graph construction, large unaligned portions (e.g. repeat arrays) of nonprimary references are clipped out to produce the final graph. Clipped chromosome graphs were subsequently merged with vg toolkit (v.1.59.0; Garrison *et al*., 2018) and the degree of sequence sharing across haplotypes in the graph was assessed with Panacus (v.0.2.3; Parmigiani *et al*., 2024a). We also calculated 31‐mer presence–absence in the genome assemblies to calculate whole‐genome sequence growth curves with Pangrowth (Parmigiani *et al*., 2024b). The same process was repeated for genic sequences and NLRs (see the ‘NLR identification’ section below).

### 6. Gene Ontology enrichment analysis

We performed Gene Ontology (GO) Enrichment Analysis using the R package topGO v.2.60.1 (Alexa & Rahnenfuhrer, 2025) and the TAIR Arabidopsis gene library (Reiser *et al*., 2024). We selected orthogroups from our analysis that included Arabidopsis genes and were either dispensable or unique in the *T. arvense* genomes. We determined enrichment of different biological processes using Fisher's exact test in topGO.

### 7. Variant calling

Variants for the sequenced reference genomes were called from the Illumina polishing libraries (see the section DNA sequencing and genome assembly above). Reads were aligned to the MN106 reference using bwa‐mem2 (v.2.2.1; Vasimuddin *et al*., 2019; alignment scripts), and alignments were postprocessed using PicardTools (https://broadinstitute.github.io/picard/). Variants were called using bcftools (v.1.20; Danecek *et al*., 2021) and filtered for minimum and maximum sequencing depth and missing data.

Genomic DNA was extracted from 95 *T. arvense* samples representing a broad geographic distribution (Dataset S5) using a modified CTAB extraction. DNA was quantified with a Quant‐iT™ PicoGreen® dsDNA kit (Life Technologies, Grand Island, NY, USA) and genotyping‐by‐sequencing (GBS) was performed as in Elshire *et al*. (2011), at the University of Wisconsin‐Madison Biotechnology Center with half‐sized reactions. Samples were digested using the *ApeK*I restriction enzyme, barcoded, and pooled for sequencing. Sample libraries were sequenced on an Illumina NovaSeq X Plus sequencer using 2 × 150‐bp reads. Samples were demultiplexed with cutadapt (v.4.9; Martin, 2011), and raw reads were aligned to the MN106 reference genome using bwa‐mem2 (v.2.21; Vasimuddin *et al*., 2019). GBS variants were called using bcftools (v.1.20; Danecek *et al*., 2021) and filtered with vcftools (v.0.1.16; Danecek *et al*., 2011) to retain sites with ≤ 30% missingness, a quality score ≥ 30, minimum depth ≥ 5, and maximum depth ≤ 50, resulting in 637 506 sites.

### 8. Principal component analysis

We selected a subset of 163 376 variants genotyped in both the GBS and the reference polishing libraries to perform principal component analysis (using the R package SNPRelate (Zheng *et al*., 2012). We further filtered these variants for minor allele frequency and removed closely linked variants (LD = 0.95), leaving 18 375 variants.

### 9. Genome binning

We binned the chromosome segments of all members of the reference pangenome panel into four categories: telomeres, ‘arms’, pericentromeres, and centromeres (Dataset S6). Centromere classification is described above. Telomeres were defined as regions with contiguous exact matches of the canonical CCCGAAA and CCCTAAA 7‐mers (or their reverse complements). All chromosomes are (sub)metacentric and have both 5′ and 3′ gene‐dense ‘arms’ and repeat‐rich pericentromeric regions relative to the centromere. Therefore, for all chromosomes, regions proximate to the telomeres can be classified as the arms, while both 5′ and 3′ regions proximate to the centromeres can be classified as pericentromeric. Given this logic, we used four 2‐Mb intervals per chromosome as ‘training’ regions. Two of these were 50 kb (‘telomere buffer’) from the nearest telomere terminus and two were 1 Mb (‘centromere buffer’) from the nearest annotated centromere region. In total, we produced 28 pericentromere and 28 arm training intervals per genome. The telomeres and centromeres were masked, and then, the remaining genomic sequence was windowed into 50‐kb‐overlapping 1‐Mb ‘test’ intervals. The relative abundance of genes, Ty1, Ty3, and other repeats were calculated for each training and test interval. The entire test set of windows was then classified into ‘arm’ or ‘pericentromere’ by the R function neuralnet, implemented in the neuralnet package (Fritsch *et al*., 2019) using default parameters. Any interval with an assignment probability <0.75 was set to ‘ambiguous’.

### 10. *F*_ST_ analysis

We used a machine‐learning approach to classify the genome into ‘chromosome arms’, ‘pericentromere’, and ‘centromere’ based on their genic and repetitive element content. The boundaries of these regions were then used with SNPRelate (Zheng *et al*., 2012) to extract SNPs subsets of the 163 376 variants in GBS and reference samples above that were only from gene‐rich or gene‐poor regions. We then calculated *F*_ST_ according to Hudson *et al*. (1992) separately for each chromosome using the R package KRIS v.1.1.6 (Chaichoompu *et al*., 2018).

### 11. NLR identification

We identified nucleotide‐binding site leucine‐rich repeat (NBS‐LRR) genes structurally in *Thlaspi arvense* using HMMER v.3.4 (Eddy, 2011) with default settings by searching protein sequences against the Pfam NBS‐LRR family (Pfam ID PF00931) raw hidden Markov model. For sequences with e‐values above the HMMER default inclusion threshold (0.01), we then batch‐searched the NCBI Conserved Domain Database (CDD) using BLAST (https://www.ncbi.nlm.nih.gov/Structure/bwrpsb/bwrpsb.cgi; Altschul *et al*., 1990; Camacho *et al*., 2008), downloaded data as ‘full’ (including both query and domain definitions), and for downstream analysis retained only the primary transcripts of proteins containing a complete NB‐ARC family or superfamily domain. Canonical NLRs contain a nucleotide binding domain and a leucine‐rich repeat sequence, with variable specialized N‐ and C‐terminal domains. We categorized pennycress NLR genes depending on the presence of three important N‐terminal domains: Toll/Interleukin receptor (TIR), coiled‐coil (CC), and RPW8 (RR8). With the same pipeline, we recovered 158 NLR genes from the *Arabidopsis thaliana* reference accession TAIR11, consistent with the lower end of literature estimates (Van de Weyer *et al*., 2019; Barragan & Weigel, 2021), which suggests that our methods are likely conservative but accurate. The distribution of core, dispensable, and unique orthogroups was similar regardless of whether orthogroups were present in Arabidopsis (present: 47 core, 33 core single‐copy, 11 variable, 3 unique; absent: 45 core, 35 core single‐copy, 21 variable, 1 unique). We also identified putative integrated domains from our CDD BLAST hits from both a stringent (Landry *et al*., 2023) and a permissive (Zeng *et al*., 2025) list of angiosperm NLR integrated domains.

We used custom R scripts (https://github.com/KevinABird/PennyPan) to extract phylogenetically hierarchical orthogroups containing NLRs and describe NLR clustering. Clusters were defined as at least two genes annotated as NLRs within no > 50 kb of each other. Syntenic blocks were identified using GENESPACE (as described previously). To describe synteny of NLR genes, we first extracted all blocks of synteny between the *Arabidopsis thaliana* TAIR11 genome and each of the seven *pennycress* genomes, calculated the total number of syntenic blocks for each *pennycress* genome relative to TAIR11, and determined how many syntenic blocks that overlapped an NLR cluster for each *pennycress* genome also overlapped an NLR cluster in TAIR11. To describe orthogroup synteny, we used the ‘.synHits’ output from GENESPACE and queried whether the syntenic hits for each gene were in the same or different orthogroups, and performed a chi‐squared test for each genome comparing NLR genes to non‐NLR genes. Variability in NLR genes was calculated using per‐site Shannon entropy according to the methods of Prigozhin and Krasileva (2021): NLR peptide sequences were aligned with mafft (Katoh & Standley, 2013) and per‐site Shannon entropy was calculated using R scripts from Prigozhin and Krasileva (2021). Alignments containing at least 10 peptides with a per‐site entropy of at least 1.5 were considered ‘high‐variability NLRs’ (hvNLRs).

NLR sequence content saturation was calculated by identifying 31‐mers shared by the seven accessions across NLR sequences and using these counts to generate growth curves with Pangrowth (Parmigiani *et al*., 2024b). Results from NLR analyses were compared against results for all genic regions and whole‐genome assemblies.

## III. Results

### 1. Updated genome assemblies reveal compartmentalized genome architecture

**Fig. 1:**
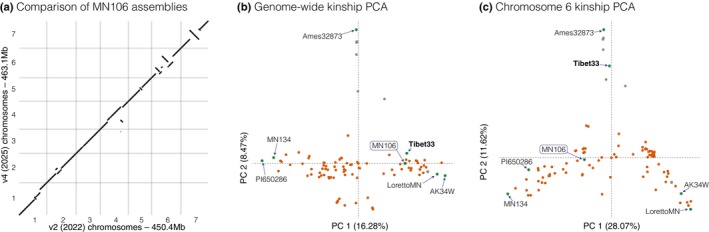
Genomic and sample diversity context for an updated MN106 reference genome. (a) Minimap2 unique best alignments (mapQ = 60) of 145 577 nonoverlapping 250‐bp ‘windowed’ v2 sequences aligned to the v4 MN106 assembly show significant improvements in v4 version. For example, a large block of Chromosome 1 is missing in v2 and inversions or translocations are present within all pericentromeres except that of Chromosome 3. Principal component decomposition of kinship genomic relationship matrices calculated genome‐wide (b) and using only Chromosome 6 (c) show consistent patterns of relatedness among six of the seven reference genotypes. However, Tibet33 clusters tightly with the Armenian samples (gray points) in Chromosome 6, potentially indicating a chromosome‐scale introgression. PCA, principal component analysis.

While the existing ‘v2’ MN106 pennycress reference genome (Nunn *et al*., 2022) has served as a valuable resource, it also has a conspicuously large ‘bottom drawer’ of 75 Mb unanchored sequence (14.3% of the genome). There were also high levels of duplicated sequence, and optical map scaffolding can introduce false contig joins. As such, we opted to assemble a new ‘v4’ MN016 reference with 88.35× PacBio HiFi (mean read length = 15 194 bp), which was subsequently scaffolded with 91.4× Omni‐C and polished with 49.1 × 2 × 150 Illumina reads. Combined, these methods produced one of the highest quality plant genome assemblies available to date. Assembly required a total of only five contig joins to form the seven chromosomes, no sequence remained in bottom drawer contigs, all 14 chromosome termini were capped with long and likely complete putative telomere sequence, and four chromosomes were single‐contig gapless (‘T2T’). Comparison between v2 and v4 assemblies revealed many major structural differences, including three large (> 1 Mb in length) translocations (total = 24.7 Mb) and 13 large inversions (total = 58.6 Mb; Fig. 1a), none of which corresponded to contig break positions in v4 but many of which colocated with contig breaks in v2. These large likely artifactual inconsistencies cover > 18.5% of the seven v2 chromosomes. Generally, structural differences between the two genome versions are aggregated near putative centromeric positions, which is often the case in optical scaffolded assemblies, and also include an obvious 7.93 Mb ‘deletion’ where the centromere of Chromosome 1 should reside.

We complemented the v4 assembly with thorough annotations. Repeats were annotated using panEDTA in EDTA2 v.2.2.1 (Ou *et al*., 2019, 2024), telomeres by clustering of putative plant telomeric repeats, and centromeres by TRASH (Wlodzimierz *et al*., 2023a) and CentIER (Xu *et al*., 2024, to be described later). Protein‐coding genes were annotated with our ‘integrative’ approach that combines transcriptome, *ab initio* and homology support. Transcriptomes were built from 296 M RNA‐sequencing Illumina paired 2 × 150 reads and 8.7 M full‐length transcript sequencing from a pooled PacBio ISO‐seq library, *ab initio*, and homology support (see the Materials and Methods section, Dataset S2). While the v2 annotation was of fairly high quality (BUSCO = 98.7, 92% single‐copy), our v4 represents a substantial improvement where protein‐coding genes capture 99.3% of embryophyte, and 100% of eukaryote, BUSCO genes (Seppey *et al*., 2019; Simão *et al*., 2015).

Initial exploration of gene and repeat density revealed a structure typical of the *Brassicaceae* family and other angiosperms: gene content was mostly confined to dense regions in the chromosome ‘arms’ (20–23% genes, 19–26% repeats) while repeat sequences predominated in the ‘pericentromeric’ regions bounding the centromeres (0.9–1.2% genes, 85–87% repeats; Fig. S1). However, unlike the closely related *Brassica* and more distant *Arabidopsis* genomes that have small pericentromeres relative to chromosome arms, pennycress genomes are dominated by expansive pericentromeres that span 297–327 Mb and make up 65–70% of the entire genome (Fig. S1). This conspicuous structure allowed us to use a neural network approach to classify the genome sequences based on gene density, repeat density, and relative abundance of repeat types where sequence proximate to telomeres and centromeres served as training sets to bin genomic windows into ‘chromosome arms’ and ‘pericentromeres’, respectively (Dataset S6). This classification revealed a highly compartmentalized genome structure: small, gene‐rich, chromosome arms (as gene‐dense as the compact *Arabidopsis thaliana* genome) and large, gene‐poor, and repeat‐rich pericentromeric blocks (Fig. S1). We then explored how this compartmentalized genome architecture shapes variation at the gene, sequence, and structural levels in the pennycress pangenome.

### 2. Genome compartmentalization preserves gene content despite extensive genome‐wide sequence variation

To form a foundation for pennycress pangenomics, we selected six genotypes in addition to MN106 that spanned key breeding samples and corners of native genetic diversity, including two accessions that are foundational for North American breeding programs (‘MN134’, ‘LorettoMN’) and others with origins from Alaska (‘AK34W’), Tibet (‘Tibet33’), Germany (‘PI650286’), and Armenia (‘Ames32873’). Exploration of SNPs for 95 diverse GBS pennycress lines and the polishing libraries of these seven genotypes confirmed that MN106, and these six other key lines capture the majority of diversity across the species (Fig. 1b). As previously noted (Frels *et al*., 2019; Nunn *et al*., 2022; Contreras‐Garrido *et al*., 2024), samples from Armenia were particularly diverged. Within‐chromosome clustering by genetic relatedness matrices also revealed some major discrepancies indicative of large‐scale chromosomal introgressions. For example, ‘Tibet33’ is closely related to the Armenian group only on Chromosome 6, but the other genetic backgrounds on all other chromosomes (Fig. 1c), suggesting the effect of chromosome‐scale introgression between deeply diverged subpopulations.

To dissect the genetic basis of divergence among these key genotypes, we assembled and annotated genomes for the six genotypes in a near‐identical manner to, and achieved similar quality statistics as, MN106 (described above; Table 1). Our previous work (Lovell *et al*., 2018, 2021; Brůna *et al*., 2026) has shown that despite very high individual quality, independent annotations even with identical methods can artificially inflate gene PAV among pangenome members. We therefore applied a second ‘harmonization’ round of protein‐coding gene model annotation where high‐confidence peptides predicted across all genomes were projected onto each individual annotation and these homology‐supported models replaced the original model if the resulting annotation had a higher score (see the Materials and Methods section) or were added to the annotation if no independent gene model existed at the given locus. The result of the harmonization round was an average of 64 genes removed, 95 added, and 89 modified across the seven annotations (Dataset S3). Overall, this approach produced between 27 470 and 28 765 high‐accuracy gene models per accession (all Eukaryote BUSCO = 100%).

**Table 1:** Genome annotation, assembly, and structure information across the seven pennycress (*Thlaspi arvense*) genomes.

	MN106	Ames32873	AK34W	LorettoMN	MN134	PI650286	Tibet33
Genome size (Mb)	463.07	460.15	456.03	455.92	461.65	466.53	456.74
HiFi coverage	88.35×	70.23×	73.80×	79.48×	73.15×	71.24×	73.56×
Contig N50 (Mb)	64.8	32.0	58.8	58.7	60.5	59.9	59.9
No. of genes	27 768	27 858	28 126	27 479	27 878	28 165	27 928
Telomeres (*n*, Kb)	14, 38.5	14, 31.6	14, 29.6	13, 36.4	14, 31.8	14, 29.6	12, 23.6
Chromosome arms (Mb, % genes)	130.5, 23.0	139.4, 21.8	152.3, 20.6	153.8, 20.1	142.0, 21.6	158.5, 19.9	141.9, 21.7
Pericentromeres (Mb, % repeats)	327.6, 85.1	308.1, 85.0	299.2, 87.1	297.1, 87.2	314.5, 86.2	303.5, 87.8	308.4, 85.3
Centromeres (*n*, Mb)	8, 4.9*	7, 12.6	7, 4.5	7, 4.9	7, 5.1	7, 4.5	7, 6.4

* Two equally computationally supported centromeric repeats exist on Chromosome 6 within genotype MN106; likely only one is recognized by the plant, but each is syntenic to the common centromere (left) or Armenia‐only centromere (right).

**Fig. 2:**
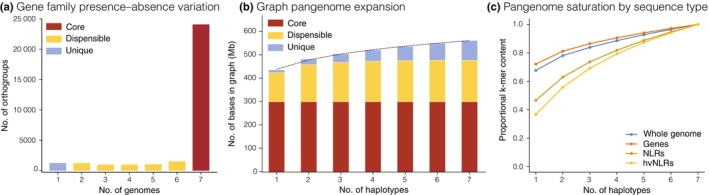
Pangenome variation in pennycress. (a) The amount of sequence in the genome graph that is shared by different numbers of haplotypes (assemblies). This figure only represents sequence in the final graph and does not contain sequences ‘clipped out’. (b) Breakdown of core, dispensable, and unique orthogroups among our seven genomes. (c) Growth curves describing patterns of 31‐mer sharing across assemblies; a steeper curve indicates a smaller degree of k‐mer sharing among assemblies for that sequence class.

To assess pangenomic variation in pennycress, we first created a pangenome graph with Minigraph‐Cactus (v.2.9.0; Hickey *et al*., 2024) using MN106 as the primary reference (Fig. 2a). On average, 95% of each assembly was retained in the final graph, which was 561.5 Mb in length and comprised 18 000 685 nodes and 24 228 161 edges. The core pangenome, or sequences in the graph shared by all haplotypes, was 300.0 Mb (*c*. 53%) in length. Of the remaining dispensable graph, 176.7 Mb (*c*. 31%) of sequence was present in two to six haplotypes, and the remaining 84.8 Mb (*c*. 15%) of sequence was unique to a single haplotype. Although our pangenome does not capture all possible sequence variation, the 1.254 decay parameter of the growth curve suggests that it captures the intended diversity at the base level (Parmigiani *et al*., 2024b).

We next queried the extent of PAV at the gene family level using 27 226 hierarchical orthogroups identified by the phylogenetic orthology inference method Orthofinder2 (Emms & Kelly, 2019), including *Arabidopsis thaliana* for comparative and additional annotation purposes (Fig. 2b; Dataset S7). Although only 53% of the total DNA sequence was identified as core, 80.3% of the orthogroups (24 077; 172 630 genes, 88.4%) were core, and 91% of core orthogroups (20 985; 146 895 genes, 75.3%) were uniformly single‐copy across all seven assemblies. For the remaining orthogroups, 4666 (16%; 20 968 genes, 10.7%) were present in two to six genomes, and 1242 (4%; 1604 genes, 0.08%) were unique to a single genome. The discrepancy between orthogroup PAV and variable sequence in the genome graph is substantially larger than reported in other recent pangenomes, perhaps indicating that our efforts to harmonize annotations across genomes effectively reduced artifactual PAV.

We also applied a kmer‐based approach to describe the amount of core and dispensable sequence (Fig. 2c), and found that sequence sharing in genic regions exhibits a similar pattern to the orthogroup‐based analysis. However, it should be noted that known highly variable genes like NLRs (to be described later) do show substantially more sequence PAV than the full genome and genic sequences. The disparity between low PAV in the highly syntenic genic regions and higher genome‐wide DNA sequence variation suggests that pericentromeric compartments are more tolerant of large‐scale structural and sequence variation than gene‐dense regions. A consequence of this compartmentalization is that although the pangenome may not capture all sequence variation in pennycress, the high sequence sharing in genic regions indicates that it does contain the vast majority of important functional variation relevant to crop improvement.

### 3. Pericentromeric genome architecture permits extensive structural variation that may constrain gene flow

**Fig. 3:**
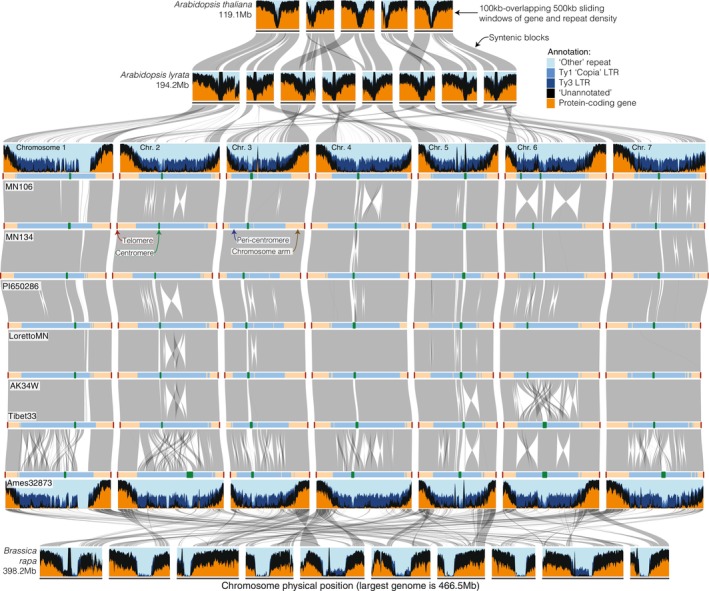
Macrosynteny and genome structure across the Brassicaceae. Horizontal blue/black/orange bands represent the chromosomes of *Arabidopsis thaliana, A. lyrata*, *Thlaspi arvense* genotypes (MN106, MN134, PI650286, LorettoMN, AK34W, Tibet33, and Ames32873), and *Brassica rapa* (top to bottom). Chromosomes are ordered by their number from left to right. Colors represent genomic content binned hierarchically in sliding windows (400‐kb‐overlapping 500‐kb) as follows: (1) within a gene annotation (including intron and untranslated region (UTR), orange); (2) within ethylenediaminetetraacetic acid‐annotated repeats were categorized as Ty3; (3) Ty1 (copia); (4) within another repeat category; or (5) un‐annotated. Gray bands are sequence‐based syntenic blocks between each pair of genomes. Pennycress and *B. rapa* are phylogenetically proximate – both belonging to the *Brassicodae* supertribe – yet they exhibit reduced synteny, due in part to genome reshuffling in *B. rapa* following a whole‐genome triplication event. The seven pennycress genome assemblies (horizontal bars) are binned into TRASH‐defined centromeres (orange), pericentromeres (dark blue), chromosome arms (light blue), and telomeres (dark red). The colors along the chromosome segments scale physically with the size of the bin, except that centromeres and telomeres have a 1‐pt buffer to make it easier to see these typically small regions. Each genome is connected to its neighbor by gray polygons that represent sequence‐based syntenic blocks. Plots, genomic bins, and syntenic blocks were built with DEEPSPACE (github.com/jtlovell/DEEPSPACE).

Variation across the genotypes sampled within our pennycress pangenome is characterized by distinct evolutionary regimes between the genome compartments: chromosome arms are highly syntenic, while the pericentromeres show extensive structural rearrangements (SVs; Fig. 3). Similar to our SNP‐based clustering, SVs were most common between the deeply diverged Armenian Ames32873 genome and the others. For example, SyRI classifications of alignments revealed that the five genomes excluding Tibet33 (to be described later) were syntenic to MN106 over more than 330 Mb (75%), but in Ames32873 only 122 Mb of sequence (24%) was syntenic to MN106 (Fig. S2A,B), mainly attributable to unalignable sequence (classified as either ‘highly diverged regions’ (HDRs) or unaligned regions; Goel *et al*., 2019). These regions are most likely complex nested genomic rearrangements (Fig. 3). In contrast to consistent genome‐wide divergence relative to the Armenian genome, the genome‐wide synteny tracking revealed high structural similarity between Ames32873 and Tibet33 for Chromosome 6 (Fig. 3) but similar structures between Tibet33 and the other genomes on the other chromosomes. These SVs are consistent with SNP‐based evidence for a single chromosome‐scale introgression (Fig. 1C).

Overall, the massive level of complex structural variation in the pericentromeric regions stands in stark contrast to the largely conserved genomic content in the subtelomeric chromosome arms and illustrates how the highly compartmentalized genome architecture results in some regions of the genome being more permissive of structural variation in pennycress. This structural variation may have important implications for genome‐wide patterns of population divergence. Structural variation is not just a form of genetic diversity but also creates complications in meiosis that reshape recombination and gene flow within and between species. This has been widely noted for inversions (e.g. Rieseberg *et al*., 1999; Noor *et al*., 2001; Rieseberg, 2001; Kirkpatrick and Barton, 2006; Navarro and Barton, 2003; Noor *et al*., 2007; Coughlan and Willis, (2018), but is increasingly appreciated for other forms of rearrangements, such as dysploid rearrangements (Beaudry *et al*., 2022) and centromere repositioning (Steckenborn & Marques, 2025). We therefore sought to assess whether large rearrangements within the centromeric and gene‐poor regions of the Armenian accession affect gene flow in pennycress. For this, we calculated pairwise *F*_ST_ (Hudson *et al*., 1992) from our SNP data between Armenian and non‐Armenian accessions to assess patterns of differentiation between gene‐rich and gene‐poor regions. Across all chromosomes, we found *F*_*ST*_ to be higher in the gene‐poor, more rearranged pericentromeric regions (median *F*_*ST*_ pericentromere = 0.54, chromosome arms = 0.48, Wilcoxon rank sum test *P* = 0.01; Fig. S3). Features of the pericentric compartment also likely play a role: Both the reduced efficacy of selection and the lower recombination rates of these regions are known to increase *F*_*ST*_. Structural rearrangements in these regions likely further reduce the potential for between‐population recombination and may therefore act as a barrier to gene flow, especially in these regions of the genome.

### 4. Centromeric regions are highly rearranged, vary in sequence, and are repositioned genome‐wide between populations

Synteny analysis revealed extensive rearrangement in centromeric regions, especially relative to the Armenian accession. However, it remained unclear whether this variation affected the centromeres, which are essential for meiosis and chromosome pairing, or merely the highly repetitive pericentromeric regions more likely to experience relaxed selection.

We sought to better characterize the contents of repeat‐rich pericentromeric and centromeric regions by identifying known pennycress centromeric satellite repeats and regions using TRASH (Wlodzimierz *et al*., 2023a) and CentIER (Xu *et al*., 2024). TRASH, which discovers repeats from regions with high kmer repetitiveness, identified five putative satellites ranging in size from 73 bp to 648 bp that constituted 3.1%–5.6% of total genome content and were concentrated in regions containing 50%–80% satellite sequence per 1‐kb window (Dataset S8; Fig. S4). The most abundant satellite (1.7–3.1% of total content), a 166‐bp sequence, occurred on all chromosomes in all accessions and was identical to a satellite previously identified from a pennycress accession from Brno, Czech Republic using FISH of sequences derived from BAC clones (Bayat *et al*., 2021). Its abundance and wide distribution suggest it may be ancestral in pennycress. Putative centromeric regions identified by CentIER showed only imperfect overlap with regions enriched for this FISH‐validated satellite. We therefore designated the approximate location of the centromere as the largest contiguous cluster of repeats within 100 kb of each other of either the 166‐bp or the 73‐bp satellite sequence (which was abundant on Chromosome 5 in all accessions).

**Fig. 4:**
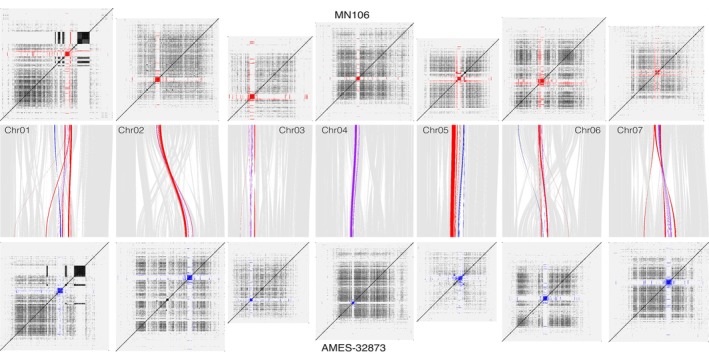
Synteny between centromeric satellite repeat regions in pennycress ‘MN106’ (top, red) and pennycress ‘Ames 32873’ (bottom, blue). Chromosome panels (top and bottom): self‐sequence mapping 24‐mers (dark regions indicate repetitive sequence). Middle panel: gray ribbons indicate background sequence synteny; red and blue ribbons indicate synteny for satellite sequences in the largest contiguous block of satellite repeats on a chromosome.

Both centromere repeat structure and position varied across accessions. In particular, the Armenian line (Ames32873) had extensive rearrangements in all centromeric regions and more total putative centromeric satellite sequence (5.6% of total sequence, compared with 3.1%–4.3% in other accessions; Fig. 4), and when inferred centromeric regions were aligned to themselves, variation in repeat structure was apparent, both within and between genomes. For example, the bounding 100 kb of alignable sequence adjacent to putative centromeres is completely syntenic between Ames32873 and MN106 only on Chromosome 4. Conversely, the syntenic position and bounding sequence identity are completely different between centromeres of Chromosomes 1 and 6. These results strongly suggest that a consequence of the repeat‐rich pericentromeric architecture is ongoing evolution of centromere structure and genomic positioning (Fig. 4). This centromeric variation may underlie incompatibilities that could impact breeding efficiency between deeply diverged gene pools.

### 5. Rapid evolution of pathogen resistance genes in the dispensable genome

#### III..1 NLR immune gene clusters evolve by high gene turnover but are positionally conserved within and between species

Although the pangenome growth curves suggest that the gene‐dense portion of the genome is comparatively conserved (Fig. 1c), the presence of dispensable and unique genes reflects variation in content and potentially in evolutionary rates. To characterize this variation, we performed GO Enrichment Analysis on Arabidopsis genes in orthogroups annotated as either dispensable or unique in pennycress. The dispensable genome (‘shell’) was enriched for genes annotated as involved in cell–cell signaling (*P* = 6.6 × 10^−12^, Fisher's exact test), and unique genes (‘private’) were enriched for defense response (*P* = 9.4 × 10^−6^, Fisher's exact test). The enrichment for defense‐related genes in highly variable portions of the genome suggests that pangenomic analyses could benefit pest and pathogen resistance, a central breeding target in pennycress (Basnet & Ellison, 2024). In addition, characterizing turnover in the dispensable genome allowed us to explore how our broader patterns of synteny in the gene‐dense region interacted with rapid evolution. We therefore focused on annotating and describing the evolution of a major class of disease‐resistance genes.

NLR genes are a major group of disease‐resistance genes in plants, known to be enriched for functionally important copy number and presence absence variation in many species (Barragan & Weigel, 2021; Lovell *et al*., 2021; Chou *et al*., 2023). We applied our pangenomic approach to characterize NLR diversity and aid pennycress breeding efforts to reduce vulnerability to diseases and pests, including soybean cyst nematode. We first structurally identified between 131 and 148 NLRs per accession with three canonical N‐terminal domains (Dataset S9). Analyses using kmer and orthogroup level variation demonstrate higher variability in NLR genes: NLRs have a substantially steeper growth curve than other genic sequences, indicating more PAV and NLR enrichment in the dispensable genome (Fig. 2c). Similarly, across the 132 phylogenetically hierarchical NLR orthogroups we identified, 52% were only identified in pennycress and not found in Arabidopsis Columbia‐0 (Col‐0), although Arabidopsis NLRs are also diverse (Van de Weyer *et al*., 2019). Within pennycress, NLR genes were slightly less likely to be included in core orthogroups (81% vs 85%, *P* = 0.2298, Fisher's exact test), somewhat more frequent in orthogroups present in two to six genomes (15% vs 12%, *P* = 0.1339, Fisher's exact test), and significantly less common in core orthogroups that are single‐copy in all accessions (58% vs 77%, *P* = 6.47 × 10^−7^, Fisher's exact test).

We also identified common NLR integrated domains from our CDD BLAST, based on a stringent and a permissive list of putative integrated domains. Using a stringent list of domains, integrated domains were rare in *T. arvense* NLRs, appearing in only three to five TIR‐NLRs per genome (compared with six in Arabidopsis; 2.6% of total pennycress NLRs), while a more permissive domain list found possible integrated domains in the majority of NLRs (98–109, 112 in Arabidopsis; 73.8% of total pennycress NLRs; Dataset S10). The most common domains from the stringent list were B3, found in at least two NLR genes in all *T. arvense* accessions, BRX, found in at least one NLR gene in every *T. arvense* accession, and WRKY, found in all *T. arvense* accessions except for AK34W and Tibet. From the permissive list, NACHT (50–57 per accession), C‐JID (43–51 per accession), COG4916 (38–43 per accession), and PRK13342 (21–30 per accession) were the most abundant. Perhaps reflecting that the stringent list was developed from comparing well‐studied species across the angiosperm tree, all 26 NLRs with putative integrated domains from the stringent list were in core orthogroups (21/26 in core single‐copy genes). Using the permissive list, 637 NLRs with integrated domains were core (345 core single‐copy), 78 shell, 11 cloud, and 2 private. NLRs including integrated domains are thus more likely to be members of core orthogroups than NLRs that do not include integrated domains (all NLRs: 81% core; stringent integrated domain NLRs: 100% core; permissive integrated domain NLRs: 87.5% core).

**Fig. 5:**
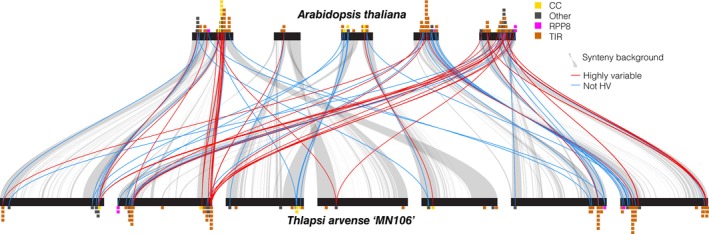
Synteny between locations of NOD‐like receptor (NLR) clusters in Arabidopsis (upper) and pennycress ‘MN106’ (lower). Stacked barplots represent NLR clusters (one block per gene), colored by N‐terminal domain. Gray ribbons indicate synteny. Red ribbons indicate synteny between highly variable NLR genes (hvNLRs), and blue ribbons indicate synteny between non‐hvNLRs.

Although it is known that NLR genes tend to group into physical clusters (van Wersch & Li, 2019), less is known about variation in the position and content of NLR clusters within and between species. Characterizing higher‐level NLR cluster evolution of this kind may help both identify candidate resistance loci in emerging crop systems and shed light on the evolutionary dynamics of these complex genomic loci. We leveraged our highly contiguous pangenome assemblies to track NLR clustering and cluster synteny within pennycress, identifying between 1 and 14 clusters of between 2 and 11 NLRs per chromosome (Table S1). The vast majority (> 90%) of pennycress NLR clusters were syntenic to regions that also contained NLR clusters in Arabidopsis (Table S2; Dataset S9). Although the physical position of NLR clusters is highly syntenic between pennycress and Arabidopsis, NLR orthogroups in syntenic clusters were similar within pennycress but differed between species, suggesting that rapid turnover erodes 1 : 1 synteny (Fig. 5). Consistent with this, NLR genes located within a cluster shared across species (pennycress NLRs with syntenic BLAST hits to Arabidopsis NLRs) were significantly less likely to be in the same orthogroup (chi‐squared with 1 df, all *P* < 0.0001). Thus, the macrosyntenic conservation of NLR clusters between Arabidopsis and pennycress does not reflect conservation of NLR gene content within these clusters.

The variation in content of syntenic NLR clusters also suggests that distinct sets of NLRs are diversifying between species, likely reflecting their unique ecologies and evolutionary pressures. We characterized NLR variability (evolutionary rates) in pennycress by calculating per‐site Shannon entropy for NLR gene‐containing orthogroups and related this to a previously identified set of highly variable NLR genes in Arabidopsis (Prigozhin and Krasileva, 2021). We identified 9% of NLR gene‐containing orthogroups (12/132), representing a total of 244 genes, as having sufficient peptide variability to count as hvNLRs. All eight orthogroups containing Arabidopsis hvNLRs were present in pennycress, and six of these varied in gene number. However, only 7 of the 12 orthogroups containing pennycress hvNLRs were present in Arabidopsis, and only three were high variability in both species. Although orthogroups containing Arabidopsis hvNLRs were more likely to contain pennycress hvNLRs, the difference was not statistically significant (chi‐squared with 1 df, *P* = 0.073), suggesting that different NLR orthogroups evolve rapidly in pennycress compared with Arabidopsis. Taken together, these analyses of NLR diversity and synteny suggest that while NLR clusters are largely positionally conserved across species, species vary greatly in their NLR content within clusters and the identity of expanding NLR orthogroups and fast‐evolving hvNLRs differs between species (Fig. 5). These results provide an example of how the compartmentalized architecture shapes patterns of variation at different scales. NLR genes are an outlier in the otherwise conserved gene‐dense chromosome arms, with high levels of gene turnover, PAV, and sequence divergence. However, the conservative genome architecture in this region limits larger structural variation, such that NLR cluster positions remain macrosyntenic, even across species boundaries, despite the extensive local variation in gene content. The identification of specific hvNLR clusters provides targets for breeding efforts to improve disease resistance. More broadly, these findings highlight a distinction between the patterns of microsyntenic turnover and 1 : 1 orthology on the one hand, and macrosyntenic conservation on the other as a feature of NLR evolution.

## IV. Discussion

In this study, we leveraged the latest long‐read sequencing technology to make seven exceptionally high‐quality genomes representing the genetic and geographic diversity of the emerging oilseed crop pennycress (*Thlaspi arvense*). Our pangenomic analyses revealed a highly compartmentalized genome architecture with gene‐dense chromosome arms and expansive pericentromeric regions (65–70% of the genome) dominated by repetitive elements. We found that this compartmentalized genome structure defined distinct evolutionary regimes: gene‐dense arms remain structurally stable and highly syntenic, even in regions of localized functional evolution, such as NLR clusters, whereas pericentromeric regions experience extensive structural rearrangement, including large inversions and genome‐wide centromere repositioning. We summarize this pattern as a ‘two‐speed’ genome with respect to structural evolution.

Interestingly, interchromosomal translocations were entirely absent and extensive pericentromeric rearrangements resulted in only minimal variation in the size of the genomes (456–467 Mb), suggesting that these processes operate within a narrow size range of DNA Kbp. Consistent with their structural stability, the vast majority of orthogroups and sequences were shared across all genomes, indicating the previously reported paucity of SNP variation in pennycress (e.g. Frels *et al*., 2019) is mirrored by low levels of pangenomic variation across much of the gene‐dense parts of the genome. By contrast, we identified extensive variation in two aspects of the pennycress pangenome: centromere structure, and the complement of immune defense NLR genes. Because both forms of variation are difficult to resolve using reference‐based approaches, our discoveries highlight the value of pangenome approaches for uncovering meaningful diversity.

### 1. Genome architecture concentrates structural variation and shapes population divergence

Our pangenomic analysis revealed extensive genomic rearrangement (*c*. 25% sequence syntenic) between the Armenian subpopulation and our six other genomes that far exceeds what has been observed in pangenomes of other species in the *Brassicaceae*, including *Arabidopsis thaliana* (70%–95% syntenic; Lian *et al*., 2024), *Brassica napus* (74–86% syntenic; Song *et al*., 2020), and *Camelina sativa* (*c*. 94% syntenic; Bird *et al*., 2025). It also exceeds other recent pangenomes like tetraploid potato (56% syntenic between two haplotypes; Sun *et al*., 2025) and *Eucalyptus* (50–53% syntenic between the most distinct subsets of species; Ferguson *et al*., 2024), despite pennycress being a relatively young diploid species (Esmailbegi *et al*., 2018). Recent reports in *Mimulus* are qualitatively similar, where large blocks of unalignable sequence in pericentromeric regions resulted in *c*. 75% synteny within a single *M. guttatus* population and *c*. 43%–54% synteny between different *Mimulus* species, but without the centromeric shifts observed in this study (Lovell *et al*., 2025). While this magnitude of genomic divergence raises the question about the proper taxonomic status of the pennycress Armenian population, there are no known reports of unsuccessful or onerous crosses between Armenian and non‐Armenian accessions (Frels *et al*., 2019) and our results suggest at least one chromosome‐scale introgression between the Armenian Ames32873 line and Tibet33. Similar signs of unidirectional gene flow from Armenian populations were seen in a recent large‐scale population genomic survey of pennycress (Wu *et al*., 2025).

Previous studies established the Armenian subpopulation as genetically diverged from other populations based on SNP data (Frels *et al*., 2019; Nunn *et al*., 2022; Contreras‐Garrido *et al*., 2024; X. Wu *et al*., 2025) and, on this basis, proposed it as a breeding source to expand genetic diversity in the species (Frels *et al*., 2019). However, because the diversity is primarily in nonfunctional pericentromeric regions and the Armenian genome did not stand out as distinct in its PAV or NLR repertoire, its use as a source of genetic diversity appears limited. Instead, this pennycress system presents an exceptional model to investigate the causes and evolutionary consequences of widespread genomic rearrangements. It also shows the importance of diverse sampling across geographical and genetic distances for uncovering structural variation, even in species with apparently low SNP diversity.

This previously unappreciated structural variation also offers insight into ongoing debates about the distribution of genetic variation across chromosomes and the forces shaping these distributions. Across taxa, repeated observations have variously found higher and lower variation in pericentromeric regions. Possible explanations for these patterns has ranged from gene density, recombination, selection, hitchhiking, domestication, and mating system (Aguadé *et al*., 1989; Kawabe *et al*., 2008; Flowers *et al*., 2012; Chen *et al*., 2022); here, we provide evidence that differences in genome architecture within pennycress genomes shape which forms of variation thatpredominate in different chromosomal regions structural rearrangements are concentrated where repeitive sequences and low recombination permit them, while gene‐dense chromosome arms maintain structural stability. While our results do not identify the underlying cause of the extensive genomic rearrangement, many likely candidates exist. TEs and other repetitive sequences are known to promote genomic instability, structural variation, and rearrangements in plant and animal genomes (Bennetzen, 2005; Balachandran *et al*., 2022). Rearrangements in pennycress are concentrated in the repeat‐rich pericentromeric and centromeric regions in which TE activity is known to be concentrated (Contreras‐Garrido *et al*., 2024). Centromere variants are also associated with meiotic drive in plants (Finseth, 2023), and the presence and abundance of drivers may vary spatially (Lindholm *et al*., 2016). Comparisons between the Armenian and non‐Armernian populations may also shed light on how genomic rearrangements contribute to speciation or limit the efficacy of back‐crosses through reduced recombination that prevents breaking LD between beneficial and deleterious variants.

Because we found evidence of extensive rearrangement in the middle of the pennycress chromosomes, we used our highly contiguous independently assembled genomes for an in‐depth query into the dynamics of complex centromeric regions. Using both previously validated and new computationally identified centromeric satellites, we estimated centromere position and structure for each chromosome across all seven assemblies, revealing that all seven centromeres were structurally rearranged and relocated in the Armenian genome, and that the repeat structures and locations also varied across non‐Armenian samples. Previous work in the *Thlaspideae* tribe has found numerous changes in centromere position and structure between genera and species, and identifies Chromosome 6 in particular as a rearrangement‐prone driver of diversification in these species (Bayat *et al*., 2021). Our results demonstrate that these processes also occur at the intraspecific level, at least in pennycress. While several recent high‐quality genomic studies have noted centromere movement within species (e.g. grape, maize, octoploid strawberry, soybean, and hexaploid wheat; Hufford *et al*., 2021; Liu *et al*., 2023; Zhao *et al*., 2023; Guo *et al*., 2025; Jin *et al*., 2025), these mostly involve a subset of chromosomes and occur through canonical epigenomic centromere repositioning without changes to the underlying DNA sequence. The observations here of genomic rearrangements repositioning multiple centromeres between subpopulations are, to our knowledge, unprecedented, although both Arabidopsis (Wlodzimierz *et al*., 2023b) and *Brassica rapa* (Ma *et al*., 2026) have recently been shown to harbor apparently adaptive centromeric variation. Pennycress presents a unique case study of genome instability and population divergence, as well as adding to a growing body of research recognizing centromeric diversity in the Brassicaceae. We are unable at present to tell whether centromeric movement occurred before the genomic rearrangements as part of canonical centromere repositioning or as a consequence of the rearrangements, or how the appearance of possible novel satellite repeats relates to this process. Future work is needed to validate centromere location with CENH profiling and formally test whether genomic rearrangements were the cause or consequence of centromere movement to shed light on possible evolutionary causes.

### 2. Distinct patterns of macro‐ and microsynteny in NLR cluster evolution and implications for resistance breeding

Immune function genes evolve rapidly in many taxa (Pancer & Cooper, 2006), and we found defense genes to be enriched in the dispensable genome. In particular, the NLR genes in plants have long been known to be highly diverse (Baggs *et al*., 2017), and vary widely in complement and structure in other *Brassicaceae* species (Van de Weyer *et al*., 2019; Jiao and Schneeberger, 2020; Zhang *et al*., 2021). Because of this known variation and because of pennycress's susceptibility to pathogens currently limiting its use in crop rotations (Basnet & Ellison, 2019), we characterized NLR diversity in our pangenome, which may accelerate breeding for disease resistance (Barragan & Weigel, 2021). Although the pennycress pangenome generally showed strong conservation of gene order and only modest PAV, variation was unevenly distributed across the genome. As in other plants, pennycress NLR genes exhibit a more ‘open’ pangenome than other genes, showing evidence of high variability and rapid evolution.

As more sequenced genomes become available, species‐specific ‘panNLRomes’ have revealed extensive variation in evolutionary rates and diversity as well as in structure, function, and genomic clustering (Van de Weyer *et al*., 2019; Lee & Chae, 2020; Prigozhin & Krasileva, 2021). Although pennycress NLRs exhibit a more open pangenome than other genes in the genome, the pennycress NLRome remains less diverse than that of several species described so far. In other species, the proportion of NLR genes identified as core ranges from 7% in wheat NLRs (Ning *et al*., 2025) to around half in cucumber (Zhang & Deng, 2026), avocado (Backer *et al*., 2025), Arabidopsis (Van de Weyer *et al*., 2019), and *Brassica napus* (Ning *et al*., 2024), while over 80% of pennycress NLRs are in core orthogroups. This suggests that although NLRs are a diverse and fast‐evolving part of the pennycress genome, they still fit the broader genome‐wide pattern of fairly low variability in genic regions. This comparative conservatism also applies to pennycress NLR integrated domains: compared with NLRs in other species, pennycress integrated domains are more likely to be in the core genome (> 80%) than those of *B. napus* (*c*. 50%, Ning *et al*., 2024), and may be rarer than in other species (*c*. 5% of wheat and Arabidopsis, 14% of *B. napus*, and 30% of cucumber NLRs include integrated domains: Van de Weyer *et al*., 2019; Ning *et al*., 2024; Ning *et al*., 2025; Zhang & Deng, 2026). This is consistent with an emerging pattern of extreme variability both within and between NLRomes: the amounts, identities, and degrees of PAV in NLR genes vary widely across plants (Baggs *et al*., 2017), as does the frequency of integrated domains (Zeng *et al*., 2025).

Recent cross‐species integrations also suggest that NLRs may maintain some level of synteny even across extremely long evolutionary timescales (Guo *et al*., 2025) and reveal the breadth of functional expansion in ancient, positionally conserved clusters (VanGessel *et al*., 2025). With the capacity to locate and resolve tandem arrays across disparate genomes, the field moves toward a more complete general picture of NLR variability at the sequence, content, and position scales and the relationship between variation at different scales. Indeed, the NLRs we identified in pennycress grouped genetically into many orthogroups and were physically concentrated in clusters distributed across the genome. Placing our pennycress NLRs into a comparative context with Arabidopsis Col‐0, we find clear evidence for NLR cluster positional conservation across species. However, rather than reflecting shared gene content, the pennycress NLR clusters we identified included several orthogroups absent from the Arabidopsis accession Col‐0 consistent with variable orthogroup content both within and between species. Peptide entropy estimates of pennycress NLR orthogroups (Prigozhin & Krasileva, 2021) further showed that high‐variability orthogroups in pennycress were largely different than the high‐variability orthogroups in Arabidopsis.

Taken together, these results support an evolutionary model in which micro‐ and macrosynteny decouple in NLR clusters, leading to distinct trajectories in cluster content and diversity across species while the surrounding genomic context remains conserved. This erosion of 1 : 1 gene synteny within positionally conserved clusters poses unique challenges to translational biology as it means winnowing down candidate genes within a cluster based on orthology may be ineffective or misleading. Functional characterization of NLR variation in pennycress could complement our genomics approach to enhance breeding.

## V. Conclusions

Genomes are not homogeneous, but rather vary in myriad features, including local recombination rate, gene and TE content, extent of heterochromatinization, and effective population size and efficacy of selection. The distributions of these features structure the degrees and kinds of variation in different genomic regions. Our results revealed pennycress as an extreme case of a compartmentalized genome architecture in which gene‐dense chromosome arms remain largely stable and conserved, while expansive gene‐poor pericentromeric regions experience extensive structural variation. In aggregate, these contrasting architecture‐dependent regimes give rise to a pattern that can be described as a ‘two‐speed’ mode of pangenomic evolution.

In a pangenomic context, our findings highlight both NLR loci and centromeric regions as classes of genomic content that undergo substantial restructuring and repositioning that would not be detectable using reference‐based approaches. In both cases, the broad functional roles are preserved, even as the underlying gene or repeat content varies extensively through turnover, rearrangement, or presence–absence variation. More generally, this study illustrates how pangenomes enable the discovery of previously elusive forms of genomic variation and provide a framework for linking genomic architecture to evolutionary dynamics, with implications for basic evolutionary biology and translational crop improvement.

## Competing interests

None declared.

## Author contributions

KAB, DJK, JLR and JTL planned and designed the research. EK, KF, KG, PB and SE provided the sample and data collection. JT and SR performed high molecular weight DNA extractions. AL, CD, JGr JGu and KB contributed to the library construction, QC, and sequencing. JWJ, RW, TB, CP and LB performed bioinformatic analyses related to genome assembly and annotation. AH, CM, KAB, JLR and JTL conducted pangenomic analyses. DJK, JGr, JS and JTL supervised the work. DJK, JCP and PPE secured funding and conceptualized the project. KAB and JLR contributed equally.

## Disclaimer

The New Phytologist Foundation remains neutral with regard to jurisdictional claims in maps and in any institutional affiliations.

## Supporting information


**Dataset S1** Genome assembly statistics.
**Dataset S2** Metadata for RNA‐ and ISO‐seq libraries used in protein‐coding gene annotations.
**Dataset S3** Gene annotation changes.

**Dataset S4** Gene model translation.

**Dataset S5** GBS accession information.
**Dataset S6** Genome classifications.

**Dataset S7** Gene family identity.
**Dataset S8** Centromere hits.
**Dataset S9** NLR genes.
**Dataset S10** NLR integrated domains.

**Fig. S1** Gene and repeat density.
**Fig. S2** Identity of alignable and unaligned sequence relative to MN106.
**Fig. S3** Population differentiation by genomic class.
**Fig. S4** Position and structure of centromeric repeat.
**Table S1** NLR gene and cluster counts by accession.
**Table S2** NLR cluster syntenic block overlap.
Please note: Wiley is not responsible for the content or functionality of any Supporting Information supplied by the authors. Any queries (other than missing material) should be directed to the *New Phytologist* Central Office.

## Data Availability

Reference genome and annotation files for *Thlaspi arvense* of AK34W (v.1.1), Ames32873 (v.1.1), LorettoMN (v.1.1), MN106 (v.4.1), MN134 (v.1.1), PI650286 (v.1.1), and Tibet 33 (v.1.1) genomes are available at https://phytozome‐next.jgi.doe.gov/pennypan/. All raw genomic and transcriptomic sequences used for assembly and annotation have been deposited in the NCBI SRA data base (Dataset S2). Raw data for GBS resequencing can be found in the NCBI SRA database under bioproject no.: PRJNA1315838.
