# Supplementary material for: Structure and sequence evolution in the pennycress (Thlaspi arvense) pangenome
Source: New Phytol. 2026 Apr 2;250(5):2723–41. doi: 10.1111/nph.71111 (PMC13150303; doi:10.1111/nph.71111)
Supplement: Supplementary file 3 — Dataset S5 GBS accession information. Dataset S6 Genome classifications. [file NPH-250-2723-s003.docx]

**Dataset S5 | GBS accession information.**

| SN | Accession | Origin |
| --- | --- | --- |
| 1 | Ames 22461 | Poland |
| 2 | Ames 23761 | Ontario, Canada |
| 3 | Ames 24499 | Former Serbia and Montenegro |
| 4 | Ames 29509 | Ohio, United States |
| 5 | Ames 29512 | Ohio, United States |
| 6 | Ames 29513 | Iowa, United States |
| 7 | Ames 29531 | North Dakota, United States |
| 8 | Ames 30933 | Magallanes, Chile |
| 9 | Ames 30982 | Iowa, United States |
| 10 | Ames 30983 | Illinois, United States |
| 11 | Ames 30984 | South Dakota, United States |
| 12 | Ames 30985 | South Dakota, United States |
| 13 | Ames 30997 | Colorado, United States |
| 14 | Ames 30998 | Colorado, United States |
| 15 | Ames 30999 | Colorado, United States |
| 16 | Ames 31000 | Colorado, United States |
| 17 | Ames 31001 | Colorado, United States |
| 18 | Ames 31002 | Colorado, United States |
| 19 | Ames 31003 | Colorado, United States |
| 20 | Ames 31004 | Colorado, United States |
| 21 | Ames 31005 | Colorado, United States |
| 22 | Ames 31006 | Colorado, United States |
| 23 | Ames 31007 | Colorado, United States |
| 24 | Ames 31008 | Colorado, United States |
| 25 | Ames 31009 | Colorado, United States |
| 26 | Ames 31010 | Colorado, United States |
| 27 | Ames 31011 | Colorado, United States |
| 28 | Ames 31012 | Colorado, United States |
| 29 | Ames 31013 | Colorado, United States |
| 30 | Ames 31014 | Colorado, United States |
| 31 | Ames 31015 | Colorado, United States |
| 32 | Ames 31016 | Colorado, United States |
| 33 | Ames 31017 | Colorado, United States |
| 34 | Ames 31018 | Colorado, United States |
| 35 | Ames 31019 | Colorado, United States |
| 36 | Ames 31020 | Colorado, United States |
| 37 | Ames 31021 | Colorado, United States |
| 38 | Ames 31022 | Colorado, United States |
| 39 | Ames 31023 | Colorado, United States |
| 40 | Ames 31024 | Colorado, United States |
| 41 | Ames 31025 | Colorado, United States |
| 42 | Ames 31026 | Colorado, United States |
| 43 | Ames 31487 | Ontario, Canada |
| 44 | Ames 31488 | Ontario, Canada |
| 45 | Ames 31489 | Saskatchewan, Canada |
| 46 | Ames 31490 | Saskatchewan, Canada |
| 47 | Ames 31491 | Saskatchewan, Canada |
| 48 | Ames 31492 | Saskatchewan, Canada |
| 49 | Ames 31493 | Saskatchewan, Canada |
| 50 | Ames 31494 | Saskatchewan, Canada |
| 51 | Ames 31495 | Saskatchewan, Canada |
| 52 | Ames 31496 | Saskatchewan, Canada |
| 53 | Ames 31497 | Saskatchewan, Canada |
| 54 | Ames 31498 | Alberta, Canada |
| 55 | Ames 31499 | British Columbia, Canada |
| 56 | Ames 31500 | Alberta, Canada |
| 57 | Ames 31501 | Manitoba, Canada |
| 58 | Ames 31502 | Manitoba, Canada |
| 59 | Ames 32239 | Colorado, United States |
| 60 | Ames 32757 | Colorado, United States |
| 61 | Ames 32867 | Armenia |
| 62 | Ames 32868 | Armenia |
| 63 | Ames 32869 | Armenia |
| 64 | Ames 32870 | Armenia |
| 65 | Ames 32871 | Armenia |
| 66 | Ames 32872 | Armenia |
| 67 | Ames 32873 | Armenia |
| 68 | Ames 33895 | Idaho, United States |
| 69 | Ames 33896 | Idaho, United States |
| 70 | Ames 33897 | Idaho, United States |
| 71 | Ames 34005 | Colorado, United States |
| 72 | Ames 34006 | Colorado, United States |
| 73 | Ames 35896 | Unknown |
| 74 | CM14 | Northeast Missouri |
| 75 | CM15 | Northeast Missouri |
| 76 | PB1 | Central Missouri |
| 77 | PB10 | Central Missouri |
| 78 | PB11 | Central Missouri |
| 79 | PB12 | Central Missouri |
| 80 | PB13 | Central Missouri |
| 81 | PB2 | Central Missouri |
| 82 | PB3 | Central Missouri |
| 83 | PB4 | Central Missouri |
| 84 | PB5 | Central Missouri |
| 85 | PB6 | Central Missouri |
| 86 | PB7 | Central Missouri |
| 87 | PB8 | Central Missouri |
| 88 | PI 633414 | Thüringen, Germany |
| 89 | PI 633415 | Sachsen, Germany |
| 90 | PI 650284 | Thüringen, Germany |
| 91 | PI 650285 | Sachsen, Germany |
| 92 | PI 650287 | Grand-Est, France |
| 93 | PI 672505 | Illinois, United States |
| 94 | PI 673443 | Illinois, United States |
| 95 | PI 677360 | Illinois, United States |
