# Supplementary material for: Structure and sequence evolution in the pennycress (Thlaspi arvense) pangenome
Source: New Phytol. 2026 Apr 2;250(5):2723–41. doi: 10.1111/nph.71111 (PMC13150303; doi:10.1111/nph.71111)
Supplement: Supplementary file 5 — Fig. S1 Gene and repeat density. Fig. S2 Identity of alignable and unaligned sequence relative to MN106. Fig. S3 Population differentiation by genomic class. Fig. S4 Position and structure of centromeric repeat. Table S1 NLR gene and cluster counts by accession. Table S2 NLR cluster syntenic block overlap. Please note: Wiley is not responsible for the content or functionality of any Supporting Information supplied by the authors. Any queries (other than missing material) should be directed to the New Phytologist Central Office. [file NPH-250-2723-s001.pdf]

New Phytologist Supporting Information
Article title: Structure and sequence evolution in the pennycress (*Thlaspi arvense*) pangenome

Authors: Kevin A. Bird, Joanna L. Rifkin, Chloee M. McLaughlin, Avril M. Harder, Pawan Basnet, Ella Katz, Tomáš Brůna, Kerrie Barry, LoriBeth Boston, Christopher Daum, Jie Guo, Anna Lipzen, Christopher Plott, Jerry W. Jenkins, Rachel Walstead, Shanmugam Rajasekar, Jayson Talag, Katherine Frels , Kathleen Greenham, Shelby Ellison, Jane Grimwood, Jeremy Schmutz, Patrick P. Edger, J. Chris Pires, John T. Lovell, Daniel J. Kliebenstein

Article acceptance date: 23 February 2026

Supplementary Figures 1
Supplementary Figure 1 | Gene and repeat density 1
Supplementary Figure 2 | Identity of alignable and unaligned sequence relative to MN106 2
Supplementary Figure 3 | Population differentiation by genomic class 2
Supplementary Figure 4 | Position and structure of centromeric repeats 3
Supplementary Tables 4
Supplementary Table 1 | NLR gene and cluster counts by accession. 4
Supplementary Table 2 | NLR cluster syntenic block overlap. 4

Supplementary Figures

Supplementary Figure 1 | Gene and repeat density

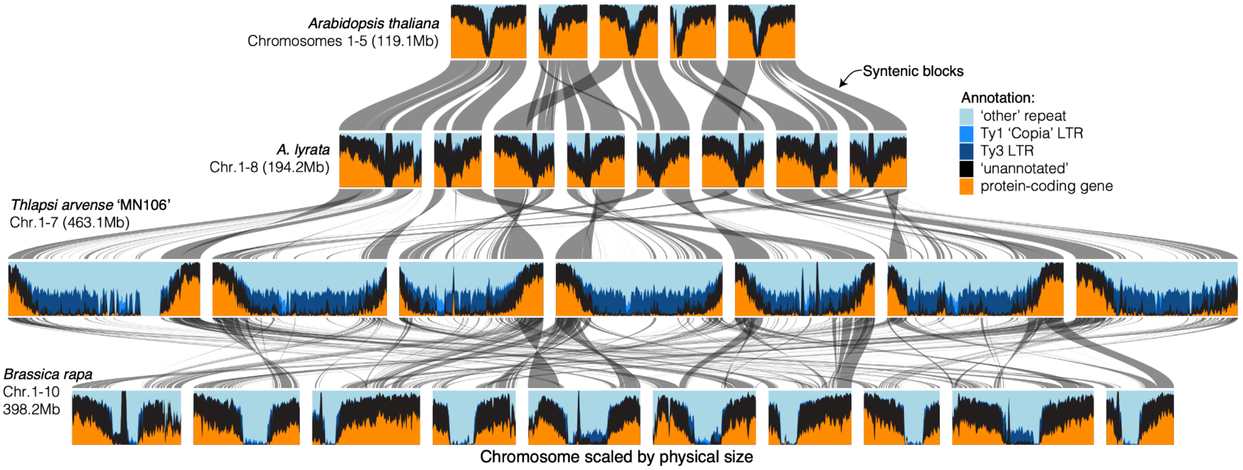

Supplementary Figure 1 | Gene and repeat density. Horizontal blue/black/orange bands represent the chromosomes of Arabidopsis thaliana, A. lyrata, *T. arvense* MN106, and Brassica rapa (top to bottom). Chromosomes are ordered by their number from left to right. Colors represent genomic content binned hierarchically in sliding windows (400kb-overlapping 500kb) as follow: (1) within a gene annotation (including intron and UTR, orange), (2) within EDTA-annotated repeats were categorized as Ty3, (3) Ty1 (copia), (4) within another repeat category, or (5) un-annotated. Grey bands are sequence-based syntenic blocks between each pair of genomes. Pennycress and *B. rapa* are phylogenetically proximate (both in Brassicodae supertribe), but have reduced synteny in part because of genome reshuffling in *B. rapa* following a whole-genome triplication event. Each genome is connected to its neighbor by grey polygons that represent sequence-based syntenic blocks. Plots, genomic bins, and syntenic blocks were built with DEEPSPACE (github.com/jtlovell/DEEPSPACE).

Supplementary Figure 2 | Identity of alignable and unaligned sequence relative to MN106

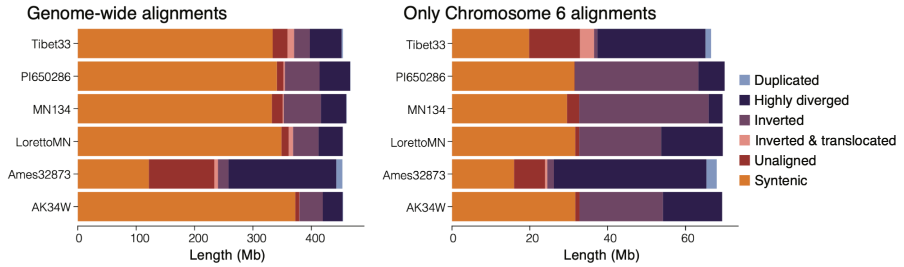

Supplementary Figure 2 | Identity of alignable and unaligned sequence relative to MN106. Genome-wide (left) and chromosome six (right) classification of sequence alignments between each genome and the MN106 reference genome from SyRI. Alignments are broken down into syntenic, not alignable to MN106, duplicated, inverted, inverted and translocated, and "highly-diverged region".

Supplementary Figure 3 | Population differentiation by genomic class

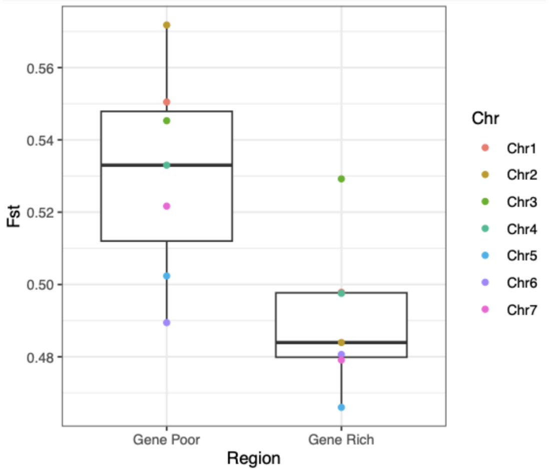

Supplementary Figure 3 | Population differentiation by genomic class. Comparison of Fst values (Hudson et al. 1992) between Armenian and non-Armenian accessions for each chromosome. Fst was calculated independently for SNPs located in gene-poor peri- and centromeric regions and gene-rich chromosome arms.

Supplementary Figure 4 | Position and structure of centromeric repeats

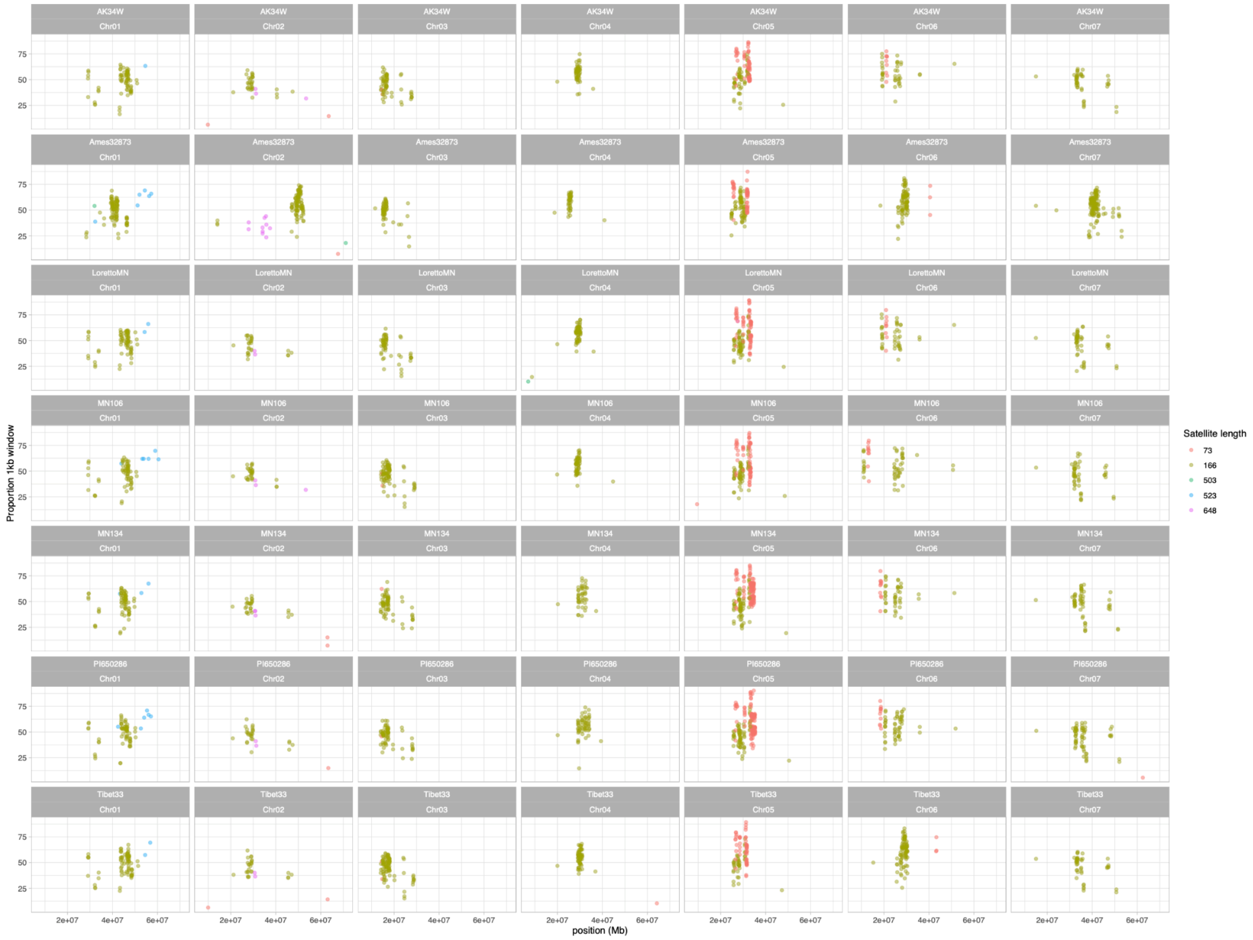

Supplementary Figure 4 | Position and structure of centromeric repeats. Content of all five repetitive sequences identified by TRASH across all seven genomes. Axes: genomic position, proportion of 1kb window consisting of putative satellite. The 166-bp and 73-bp satellites were incorporated into downstream analyses; the 523- and 503-bp sequences showed partial similarity to 16sRDNA.

Supplementary Tables

Supplementary Table 1 | NLR gene and cluster counts by accession.

Supplementary Table 1 | NLR gene and cluster counts by accession. Number of NLR genes identified, number of clusters, and cluster size for the seven pennycress genomes. Clusters were defined as at least two genes annotated as NLRs within no more than 50kb of each other.

Accession	Total Number of NLR genes identified	Number of NLR clusters	NLR cluster size range
AK34W	142	30	2-6
Ames32873	143	30	2-8
Loretto	131	28	2-6
MN106	141	28	2-7
MN134	148	28	2-11
PI650286	136	26	2-10
Tibet	145	33	2-6

Supplementary Table 2 | NLR cluster syntenic block overlap.

Supplementary Table 2 | NLR cluster syntenic block overlap. Although NLR clusters occupy only a small proportion of the genome, regions containing pennycress NLR clusters are extremely likely to be syntenic to regions containing Arabidopsis NLR clusters. Syntenic blocks between Arabidopsis and each pennycress genome were extracted from 'synHits.txt' in GENESPACE output. Overlap was calculated using custom R scripts.

Accession	Total number of syntenic blocks with Arabidopsis	Number of syntenic blocks containing an arabidopsis NLR cluster (percent of total blocks)	Base pairs containing by NLR clusters / total scaffold length (percent of total length)	NLR clusters in syntenic blocks containing arabidopsis NLR cluster / total NLR clusters (percent of total clusters)
AK34W	134	21 (15.7%)	1,099,594 / 456,034,515 (0.2%)	30/30 (100%)
Ames32873	128	18 (14.1%)	925,322 / 460,145,399 (0.2%)	29/30 (96.7%)
Loretto	133	18 (13.5%)	923,847 / 455,918,564 (0.2%)	28/28 (100%)
MN106	139	22 (15.8%)	975,682 / 455,918,564 (0.2%)	27/28 (96.4%)
MN134	140	20 (14.3%)	969,396 / 461,649,255 (0.2%)	27/28 (96.4%)
PI650286	138	20 (14.5%)	756,104 / 466,533,064 (0.16%)	24/26 (92.3%)
Tibet	128	18 (14.1)	975,908 / 456,735,805 (0.2%)	31/33 (93.9%)
